# A Clinician’s Guide to Dosing Analgesics, Anticonvulsants, and Psychotropic Medications in Continuous Renal Replacement Therapy

**DOI:** 10.1016/j.ekir.2021.05.004

**Published:** 2021-05-13

**Authors:** Rima H. Bouajram, Linda Awdishu

**Affiliations:** 1 Department of Pharmaceutical Services, University of California, San Francisco Medical Center, San Francisco, California, USA; 2 San Diego Skaggs School of Pharmacy and Pharmaceutical Sciences, University of California, La Jolla, California, USA

**Keywords:** acute kidney injury, analgesic, anticonvulsant, CRRT, dosing optimization, psychotropic

## Abstract

Acute kidney injury (AKI) requiring continuous renal replacement therapy (CRRT) is a common complication in critical illness and has a significant impact on pharmacokinetic factors determining drug exposure, including absorption, distribution, transport, metabolism, and clearance. In this review, we provide a practical guide to drug dosing considerations in critically ill patients undergoing CRRT, focusing on the most commonly used analgesic, anticonvulsant, and psychotropic medications in the clinical care of critically ill patients. A literature search was conducted to identify articles in which drug dosing was evaluated in adult patients receiving CRRT between the years 1980 and 2020. We included articles with pharmacokinetic/pharmacodynamic analyses and those that described medication clearance via CRRT. A summary of the data focused on practical pharmacokinetic and pharmacodynamic principles is presented, with recommendations for drug dosing of analgesics, anticonvulsants, and psychotropic medications. Pharmacokinetic and pharmacodynamic studies to guide drug dosing of analgesics, anticonvulsants, and psychotropic medications in critically ill patients receiving CRRT are sparse. Considering the widespread use of these medications, narrow therapeutic index of these drug classes, and risks of over- and underdosing, additional studies in patients receiving CRRT are needed to inform drug dosing.

Acute kidney injury (AKI) is a common complication in critically ill patients with various etiologies and risk factors. Admissions for AKI have increased in the past decade,^1^ compounded by COVID-19 critical illness,^2^ and have surpassed the incidence of hospitalizations from end-stage renal disease (ESRD).^3^ The varying etiologies of AKI create complex interactions when evaluating changes in drug pharmacokinetics and exposure. Continuous renal replacement therapy (CRRT) is used in approximately 5% to 10% of patients with AKI, and affects medication clearance.^4^ The optimal intensity of renal replacement therapy in critical illness is debated, but a prospective, multicenter, randomized trial demonstrated no significant impact of higher-intensity renal replacement therapy on mortality in patients with sepsis.^5^ Acute kidney injury challenges clinicians in achieving pharmacodynamic (PD) targets and predisposes patients to greater risk for medication-related adverse effects.^6^ AKI requiring CRRT increases morbidity and mortality and the contribution of under- or overdosed medication exposures to morbidity and mortality has not been well defined.^7^^,^^8^

The pathophysiologic changes in critical illness and AKI affect all pharmacokinetic factors determining drug exposure, including absorption, distribution, transport, metabolism, and clearance. This causes variability in pharmacokinetic (PK) results and fluctuations in drug concentrations, and affects pharmacodynamics. Changes in cardiac output and perfusion to organs may result in multiorgan dysfunction (MOD) and varied renal and nonrenal medication clearance.^9^^,^^10^ Modalities such as CRRT also affect PK and PD through drug removal and, to a lesser extent, alteration in nonrenal clearance.^11^^,^^12^

Despite the ever-increasing rise in adverse effects associated with medication over- and underdosing in the intensive care unit (ICU) setting,^13^, ^14^, ^15^ and particularly in patients receiving analgesics, anticonvulsants, and psychotropic medications,^16^, ^17^, ^28^ PK and PD studies to guide drug dosing in critically ill patients are sparse. Previous (2010) and current (2020) Food and Drug Administration draft guidelines recommend renal impairment studies for drugs, with some exception; however, CRRT studies are not required, and adherence to guidelines has not been routinely enforced. Pharmacokinetic analysis using modeling extrapolated from chronic kidney disease (CKD) or ESRD patients after a single dose of drug administered remains a common study technique, which may not accurately reflect drug clearance in all clinical settings and patient populations in which multiorgan dyfunction and systemic inflammatory response are prevalent.^2^^,^^12^^,^^29^ Given the limited number of new medications developed for use in the ICU setting, most drug dosing algorithms follow historic dosing recommendations that are largely based on a 1-size-fits all approach in healthy volunteers. It has been estimated that less than 20% of medications used in AKI have CRRT dosing recommendations^12^^,^^30^; gaps in knowledge to guide appropriate drug dosing in CRRT remain highly prevalent at this time.

Multiple recent reviews to date have been published on antimicrobial dosing in CRRT^31^^,^^32^ and general principles of CRRT use.^33^, ^34^, ^35^ However, a review of analgesic, anticonvulsant, and psychotropic medication dosing in CRRT is warranted.^36^^,^^37^ The purpose of this review is to provide a practical guide to the most pertinent considerations for drug dosing in critically ill patients undergoing CRRT, and summarize the literature supporting dosing recommendations for the most commonly used analgesic, anticonvulsant, and psychotropic medications in the clinical care of patients in the ICU setting.

## Methods and Data Sources

A PubMed search was conducted to identify articles where drug dosing was evaluated in the setting of CRRT between the years 1980 to 2020. Search terms included the following: *continuous renal replacement therapy, CRRT, renal replacement therapy*, *RRT, continuous venovenous hemofiltration, CVVH, continuous venovenous hemodialysis, CVVHD, continuous venovenous hemodiafiltration, CVVHDF, analgesic, sedation, antipsychotic, benzodiazepine, anticonvulsant*, *oxycodone, hydromorphone, morphine, fentanyl, methadone, propofol, dexmedetomidine, ketamine, acetaminophen, tramadol, gabapentin, pregabalin, clonidine, valproic acid, carbamazepine, levetiracetam, phenytoin, lacosamide, topiramate, lorazepam, midazolam, clonazepam, diazepam, pentobarbital, phenobarbital, buproprion, trazodone, venlafaxine, fluoxetine, paroxetine, sertraline, amitriptyline, nortriptyline, quetiapine, risperidone, and aripiprazole*. All manufacturer product information for medications listed were reviewed, and a primary literature search was performed with combinations of search terms listed.

We included articles with adult patient pharmacokinetic/pharmacodynamic (PK/PD) analysis and those that described medication clearance via CRRT for all indications. We excluded articles containing PK/PD analysis for pediatric patients and studies evaluating the use of slow (or sustained) low-efficiency daily diafiltration and slow continuous ultrafiltration. All pertinent reviews and studies were analyzed for appropriateness for inclusion. Data were summarized based on practical PK and PD principles, with tables to support drug dosing recommendations and application of principles. Dosing concepts without sufficient literature to support recommendation were excluded from the review.

## Review of Literature

A total of 18 case reports, 18 single-center studies, 9 multicenter studies, and 31 meta-analyses and review articles were evaluated. The results below summarize the most common factors influencing drug dosing in critically ill patients undergoing CRRT.

### Pharmacokinetic Changes Affecting Drug Exposure

#### Absorption

Drug absorption may be influenced by critical illness in different ways, depending on the route of medication absorption and clinical circumstances. For example, patients admitted with shock receiving vasopressors or with volume overload may have fluctuating or compromised perfusion to intestinal microvilli and subcutaneous tissues, and therefore altered absorption of enteral and subcutaneous medications.^38^ Decreased gastric motility and gastrointestinal transit time in the setting of narcotic use or vomiting, or increased transit time in the setting of diarrhea, may be present in critical illness as well and can affect drug absorption. Fluctuations in gastric pH, first-pass metabolism, intestinal p-glycoprotein activity, intestinal atrophy, and mucosal ischemia and reperfusion injury^39^ may also alter the efficacy of enteral medications. The presence of uremia may affect medication absorption by decreasing the activity of major drug transporters.^40^ Furthermore, the concurrent use of acid-suppressive medications for stress ulcer prophylaxis may alter drug absorption due to drug−drug interactions and alterations in pH-dependent solubility,^41^ which complicate estimation of drug absorption in critically ill patients.^9^ For these reasons, the administration of critical medications via the enteral route should be avoided because of the erratic and unpredictable absorption in unstable critically ill patients.

#### Distribution

The distribution of medications may be affected by the critical insult (e.g., hemorrhagic shock, liver failure, heart failure, etc.) and volume shifts during critical illness (e.g., volume overload resulting in expanded interstitial edema or serous collections in pleural and abdominal cavities, as well as decreased intravascular volume). Critically ill patients often have large obligate intake of fluids and concomitant medications to treat their underlying disease state (e.g., fluid resuscitation, antimicrobial therapies, and vasopressors). The increased volume of distribution may affect the therapeutic efficacy of hydrophilic medications and may result in greater accumulation of lipophilic medications. Decreased protein binding in the setting of hypoalbuminemia, extracellular shifts, and uremia result in altered conformational binding of medications may ultimately affect the distribution and efficacy of medications at target sites of action.^9^

#### Metabolism

In the setting of organ dysfunction, fluctuations in organ perfusion may lead to fluctuations in renal and nonrenal metabolism.^10^ In the early stages of critical illness, increases in cardiac output in the setting of stress response may result in increased medication metabolism due to increased perfusion to organs that metabolize medications. In the setting of hypoperfusion to the organs, however, organ dysfunction may develop, and decreased metabolism may occur.^9^

On a tissue and cellular level, uremia, immune function impairment, and disease states involving cytokines affect the pharmacokinetics of drugs through the regulation of expression and activity of drug-metabolizing enzymes and drug transporters.^42^ Endotoxin, interleukin-1 release, and tumor necrosis factor (TNF) release in the setting of inflammation have been thought to depress cytochrome P450 drug metabolism, affecting phase I hepatic metabolism. Effects on phase II hepatic metabolism may also be seen, but to a lesser degree.^43^ In some patient populations, such as traumatic brain injury or early burn patients, a hypermetabolic and catabolic state may occur and result in significant increase in metabolism. In combination with an acute phase stress response, the impact of metabolizing enzyme and transporting enzyme activity and cellular changes in reaction to stress response result in alterations in overall drug metabolism in critical illness.

#### Elimination

Finally, elimination of medications may be affected by critical illness. Systemic hypotension, alterations in the microcirculation, and inflammatory tissue reactions caused by the immune response can result in organ failure. Inflammatory mediators such as interleukin-6 and TNF alter cytochrome P450 drug metabolism in the liver, which may lead to decreased elimination of hepatically cleared medications.^44^^,^^45^ In general, a reduction in creatinine clearance corresponds to reductions in the clearance of renally cleared medications. In critically ill patients with AKI, fluid overload was associated with a lower serum creatinine but increased overall mortality, highlighting the limitations of using creatinine as a biomarker of kidney function.^46^ In addition, current kidney function estimating equations such as the Cockcroft−Gault, Modification of Diet in Renal Disease, Chronic Kidney Disease Epidemiology, or Jelliffe equations were not derived from or validated in the unstable, critically ill population, resulting in decreased accuracy and precision when applied to estimate kidney function during AKI.^47^ Adjustments to the Jelliffe equation for volume status have been attempted to address the impact of volume overload^46^; however, all of the estimating equations assume steady-state creatinine production, and reductions in creatinine synthesis during sepsis have been well documented.^48^ The duration of kidney failure may affect the potential for recovery of kidney function while on CRRT, as well as intrinsic drug elimination.

We have discussed in detail the pharmacokinetic impact when CRRT is initiated in the setting of AKI. However, there are circumstances in which CRRT is administered in the setting of normal renal function (e.g., for other reasons such as volume overload, severe electrolyte disturbances, or toxin clearance). Degree of drug elimination in this setting may depend partly on host intrinsic elimination, and then additionally on the CRRT prescription. The CRRT circuit may provide elimination of medications in addition to host clearance.^11^^,^^12^

### CRRT Modality, Prescription, and Advancements

#### Modality

The decision to use intermittent hemodialysis (IHD) or CRRT is usually guided by patient hemodynamics, volume removal, or risk of cerebral edema.^49^ Hemodynamically unstable patients are often placed on CRRT; however, randomized trials have not demonstrated a benefit in mortality or renal recovery.^50^, ^51^, ^52^ Continuous renal replacement therapy may be provided as continuous venovenous hemofiltration (CVVH), continuous venovenous hemodialysis (CVVHD), or continuous venovenous hemodiafiltration (CVVHDF). Each provides solute clearance and volume removal; however, the mechanisms for solute clearance may be convection or diffusion or a combination of both.

Convection is the process of hemofiltration across a semipermeable membrane, driven by a gradient in fluid hydrostatic pressures. Solutes are driven across the membrane by “solvent drag” as the solvent is forced across the membrane. In CVVH, the solute removal occurs by convection, and the major determinant of convective clearance is the ultrafiltration rate. Convective clearance is dependent on membrane pore size and the free fraction of drug. The sieving coefficient (SC) measures the ability of a drug to convectively pass through a membrane, and is calculated by the ratio of the drug concentration in the ultrafiltrate to the drug concentration in the plasma:

$$\text{SC}\;=\;{\text{Drug}}_{\text{ultrafiltrate/}}{\text{Drug}}_{\text{plasma}}$$

This value is driven primarily by solute molecular weight and membrane pore size. For solutes with an SC of 1, the magnitude of diffusive clearance equals the dialysate flow rate. In CRRT, the slow blood and dialysate flow rates always allow for equilibration of concentrations across the membrane. A drug that freely crosses the membrane, with an SC of 1, likely has low protein binding and low molecular weight. Larger molecules with an SC of less than 1 may not reach equilibrium. The “cutoff point,” which is the molecular weight of the smallest solute retained by the membrane, is an important determinant of large molecule clearance. It is defined as the molecular weight of a solute with an SC of 0.1. In the absence of a measured SC, the SC may be estimated from the medication's fraction bound (Fb) to proteins within the blood:

SC=1-Fb

A large volume of plasma is filtered across the membrane during hemofiltration. This volume is replaced by “replacement fluid” of known composition in amounts equal to or less than the total volume removed via ultrafiltration for a prescribed fluid balance (e.g., negative fluid balance). Replacement fluid is administered either pre- or postfilter, or both. In predilution CVVH, in which the replacement fluid is administered before the filter, dilution of the blood prior to filtration reduces overall clearance. The rate of drug removal can be estimated by accounting for the impact of dilution from replacement fluids (Table 1). The magnitude of pre-filter replacement fluid will lower the concentration of the drugs entering the hemofilter, thereby decreasing drug clearance. This can be estimated by using the following correction factor:

DoseofCRRT=Qeffluent∗[Qb∗(1-Hct)/Qb∗(1-Hct)+Qrf_pre]

where Qeffluent is the total effluent flow in mL/kg/hour, Qb the blood flow rate multiplied by 1-Hct to calculate plasma flow, and Qrf_pre is the rate of replacement fluid entering the blood pre-filter.

**Table 1:** Estimating clearance from different continuous renal replacement therapy modalities

Modality	Drug clearance
CVVH_pre_	CL_drug_ ~ delivered time averaged effluent rate∗SC∗ (Q_b_/Q_b_+Q_rf_)
CVVH_post_ CVVHDF	CL_drug_ ~ delivered time averaged effluent rate∗SC
CVVHD	CL_drug_ ~ delivered time averaged effluent rate∗SA

CL_drug_ = clearance of drug; CVVH_post_ = continuous venovenous hemodialysis with postfilter replacement fluid; CVVH_pre_ = continuous venovenous hemodialysis with prefilter replacement fluid; CVVHD = continous venovenous hemodialysis; CVVHDF = continuous venovenous hemodiafiltration; Q_b_ = blood flow rate; Q_rf_ = replacement fluid rate; SC = sieving coefficient.

Replacement fluid entering the blood postfilter does not dilute the plasma in the filter and therefore is not included in the correction factor. In postdilution CVVH, in which the replacement fluid is administered only postfilter, the rate of drug removal usually corresponds to the ultrafiltration flow rate multiplied by the SC (Table 1).

In CVVHD, drug clearance occurs via a simple diffusion and can be estimated based on the saturation coefficient (SA) and dialysate flow rate (Table 1). The SA is defined as the fraction of drug diffused through the CRRT membrane into the dialysate fluid. Small substances, such as urea (60 Daltons), diffuse more quickly than larger ones, such as antibiotics (300–500 Daltons). In CVVHDF, the convection is combined with diffusion, and, as a consequence, maximum clearance over the entire molecular weight spectrum is achieved. In a study by Brunet *et al.*, convective and diffusive clearance were found to be additive for small solutes, indicating that there was no significant interaction between diffusion and convection.^53^ However, for large molecules such as β-2 microglobulin, total clearance was not significantly increased when dialysate flow rates were increased, and ultrafiltration rates remained constant, indicating that convection was the predominant clearance. In CVVHDF, total clearance can be estimated by adding the ultrafiltration and dialysate flow rates and multiplying by the SC or SA (Table 1). If the replacement fluid administered is pre-filter, the calculation should be corrected for pre-dilution when appropriate (see above). Using prescribed effluent flow rates to predict drug clearance is inaccurate, as numerous studies have demonstrated that delivered clearance is lower than prescribed.^54^, ^55^, ^56^

#### Prescription

##### Hemofilters

Biocompatible, semipermeable, hollow-fiber dialyzers are the standard of care for CRRT. The hemofilter membrane “flux” is categorized by the membrane ultrafiltration coefficient (Kuf):

Kuf=(Quf/TMP)∗1/A

where Quf = ultrafiltration flow, TMP = transmembrane pressure, and A = area of the filter.High-flux hemofilters used in CRRT have larger pore sizes to allow for clearance of larger molecules. High flux is defined as an ultrafiltration coefficient (Kuf) of greater than 25, whereas low flux is less than 10. The filter lifespan has been extended due to citrate or heparin anticoagulation; however, the longer lifespan may affect performance, leading to lower clearances of antimicrobials such as gentamicin over time.^57^ In addition, the clearance of gentamicin was reduced before observing a reduction in urea clearance, demonstrating that using urea clearance is not a sensitive marker of hemofilter performance for drug clearance.^57^ The Gibbs−Donnan effect occurs when charges generated on the membrane cause retention of medications to the membrane. For example, the anionic charges on albumin in the blood will cause retention of cations such as aminoglycosides; however, the clinical significance of this effect is unclear.^58^

##### Prescribed Effluent Volume and CRRT Intensity

Kidney Disease: Improving Global Outcomes (KDIGO) clinical practice guidelines recommend delivering an effluent volume of 20 to 25 ml/kg per hour for patients with AKI on CRRT.^49^ However, additional studies are needed to determine the optimal dose in different subpopulations (i.e., sepsis, cardiac surgery, etc.). Roberts *et al.* demonstrated that, at the same CRRT intensity, there was wide variability in antibiotic concentrations, and that higher-intensity CRRT did not significantly increase the clearance of all antimicrobials but did affect vancomycin clearance.^59^^,^^60^ Overall, 15% dosing intervals (n = 40) did not achieve the antibiotic therapeutic targets, 40% did not achieve higher target concentrations, and 10% were excessive in dosing.^59^^,^^61^, ^62^, ^63^, ^64^, ^65^ An effluent volume of greater than 35 ml/kg per hour for patients on CRRT may result in increased clearance of important drugs such as antimicrobials and anticonvulsants,^66^ potentially contributing to worse outcomes observed in trials of patients on high-dose CRRT. As kidney function recovers, it is important to quantify residual kidney function to add to the estimated clearance by renal replacement therapy. As intrinsic kidney function improves, this may lead to higher clearance and risk of underdosing. The transition off of renal replacement therapy presents another period of rapidly changing clearance, placing patients at risk for dosing errors. Intrinsic kidney function can be quantified by timed urine collections for measurement of creatinine clearance.^67^ Real-time glomerular filtration rate can be measured by exogenously administering fluorescent compounds the concentrations of which can be measured by the transdermal route.^68^ Use of transdermal fluorescent glomerular filtration rate biomarkers present a promising method to address gaps in kidney function estimation using traditional biomarkers in critically ill patients. However, this technology is not yet Food and Drug Administration approved, and studies are ongoing to determine the utility of real-time glomerular filtration rate estimation in critically ill patients.^69^ A thorough evaluation of kidney function using timed urine collections and medication dose adjustment during renal recovery is imperative for ensuring that therapeutic efficacy is optimized during this transition period.

### Analgesic, Anticonvulsants, and Psychotropic Medication Properties Affecting CRRT

There are multiple medication characteristics that affect medication clearance in patients undergoing CRRT. We will discuss the most pertinent factors to consider when dosing analgesics, anticonvulsants, and psychotropic medications. Common pharmacokinetic characteristics of medications are summarized in Table 2.^70^, ^71^, ^72^, ^73^, ^74^, ^75^, ^76^, ^77^, ^78^, ^79^, ^80^, ^81^, ^82^, ^83^, ^84^, ^85^, ^86^, ^87^, ^88^, ^89^, ^90^, ^91^, ^92^, ^93^, ^94^, ^95^, ^96^, ^97^, ^98^, ^99^, ^100^, ^101^, ^102^, ^103^, ^104^, ^105^, ^106^, ^107^, ^108^, ^109^, ^110^, ^111^, ^112^, ^113^, ^114^, ^115^, ^116^, ^117^, ^118^, ^119^, ^120^, ^121^, ^122^, ^123^, ^124^, ^125^

**Table 2:** Pharmacokinetic parameters in healthy volunteers versus critical illness

Medication	MW (Daltons)	Protein binding (%)	V_d_ (L/kg)	t_1/2_ (h)	PK/PD characteristic	Excretion	Recommended adjustments in critical illness and CRRT
**Analgesics**^a^							
Oxycodone^70^	315	38	2.6	3.7	Relatively hydrophilic, evaluate efficacy using NRS/CPOT	Urine	2.5–5 mg p.o./p.f.t. q. 4–6 h p.r.n.
Hydromorphone^71^	285	8	4	2	Context-sensitive half-life, lipophilic, evaluate efficacy using NRS/CPOT	Urine, Feces	1–2 mg p.o./p.f.t. q. 4–6 h p.r.n. 0.2–0.5 mg i.v. q. 2–4 h p.r.n.
Morphine^72^^,^^73^	285	31	1	2	Relatively hydrophilic, evaluate efficacy using NRS/CPOT	Urine, Feces	5 mg p.o./p.f.t. q. 4–6 h p.r.n. 0.5–2 mg i.v. q. 2–6 h p.r.n.
Fentanyl^74^	336	87	4	2	Context-sensitive half-life, lipophilic, evaluate efficacy using NRS/CPOT	Urine 75%, Feces 9%	12.5–50 μg i.v. q. 15–60 min p.r.n.
Methadone^75^	309	85	1	8	Context-sensitive half-life, lipophilic, evaluate efficacy using NRS/CPOT	Urine	5–10 mg p.o./p.f.t. q. 6–12 h p.r.n. 2.5–5 mg i.v. q. 6–12 h p.r.n.
**Anticonvulsants**							
Valproic acid^76^, ^77^, ^78^	144	90	0.14	6	Lipophilic; evaluate free valproic acid concentrations (Note: total valproic acid concentrations may be misleading)	Urine	Seizures: 10–15 mg/kg per day Status epilepticus: 20–40 mg/kg (max. 3 g) (Divided dosing: i.v. q. 6 h, p.o./p.f.t. q. 6–8 h)
Carbamazepine^79^, ^80^, ^81^	236	27	0.6	12	Lipophilic	Urine 72%, feces 28%	Seizures: 2–3 mg/kg per day p.o./p.f.t. q. 6–12 h
Levetiracetam^82^, ^83^, ^84^, ^85^	170	<10	0.5	6	Lipophilic	Urine	Seizures: 750–1000 mg q 12 h
Phenytoin^86^^,^^87^	252	90	0.5	12	Lipophilic; evaluate free phenytoin concentrations	Urine	Seizures: 15 mg/kg (max. 2 g) i.v. loading dose divided q. 8–12 h, 4–7 mg/kg per day divided q. 6–12 h
Lacosamide^88^, ^89^, ^90^	250	<15	0.6	13	Lipophilic	Urine 95%, feces <0.5%	Seizures: 100 mg i.v./p.o./p.f.t. q. 12 h Status epilepticus: 400 mg i.v. loading dose, followed by 200 mg i.v. q. 12 h
Topiramate^91^^,^^92^^b^	339	15	0.6	19	Lipophilic	Urine	Seizures: 50 mg p.o./p.f.t. q. 24 h Status epilepticus: 200 mg p.o./p.f.t. Q12 h
Lorazepam^93^^,^^94^	321	85	1.3	12	Context-sensitive half-life, lipophilic, evaluate RASS	Urine 88%, feces 7%	Anxiety: 0.5–2 mg i.v./p.o. q. 4–6 h p.r.n. Seizures: 2–4 mg i.v. q. 3–4 min p.r.n.
Midazolam^94^^,^^95^	326	97	1	3	Context-sensitive half-life, lipophilic, evaluate RASS	Urine 90%, feces 10%	Anxiety/sedation: 0.5–5mg i.v. Q15 min p.r.n.; avoid continuous sedation, if needed for mechanical ventilation start 0.01 mg/kg per hour Status epilepticus: 0.2 mg/kg i.v. q. 3–5 min, 0.05–2 mg/kg per hour continuous i.v. infusion
Clonazepam^96^	316	85	1.5	17	Context-sensitive half–life, lipophilic, evaluate RASS	Urine	0.25 mg p.o./p.f.t. q. 12–24 h
Diazepam^97^	285	96	1	60	Context-sensitive half-life, lipophilic, evaluate RASS	Urine	Anxiety/sedation: 2–10 mg i.v./p.o./p.f.t. q. 4–6 h p.r.n. Status epilepticus: 5–10 mg i.v. q. 3–5 min p.r.n.
Pentobarbital^98^, ^99^, ^100^	226	20	1	22	Context-sensitive half-life, lipophilic, pentobarbital concentrations	Urine	Sedation: 100 mg i.v. q. 3–5 min Seizures: 5–13 mg/kg bolus, followed by 0.5–5 mg continuous infusion
Phenobarbital^100^, ^101^, ^102^, ^103^	232	48	0.6	79	Lipophilic, monitor RASS, phenobarbal concentrations	Urine 25%	Sedation: 30–120 mg/d i.v./p.o. in 2–3 divided doses Seizures: 2 mg/kg per day i.v./p.o. in 2–3 divided doses Status epilepticus: 15–20 mg/kg i.v.
Gabapentin^104^^,^^105^	171	<3	0.8^c^	5	Lipophilic, monitor RASS	Urine	100–300 mg p.o./p.f.t. q. 12 h
Pregabalin^106^	159	0	0.5	6.3	Lipophilic, monitor RASS	Urine 90%	25–50 mg IR p.o./p.f.t. q. 8 h
**Additional Sedatives and Psychotropic Medications**^d^							
Propofol^107^	178	97	2	4	Context-sensitive half-life, lipophilic, monitor RASS	Urine 88%, feces <2%	5–75 μg/kg/min continuous infusion
Dexmedetomidine^108^	200	94	1.7^c^	2	Context-sensitive half-life, lipophilic, monitor RASS	Urine 95%, feces 4%	0.2–1.5 μg/kg continuous infusion
Ketamine^109^	238	27	2.4	0.25	Context-sensitive half-life, lipophilic, monitor RASS	Urine 91%, feces 3%	Sedation: 0.5–2 mg/kg bolus, 0.2–2.5 mg/kg per hour continuous infusion
Acetaminophen^110^^,^^111^	151	10	1	2	Hydrophilic, monitor NRS/CPOT	Urine <5%	325–1000 mg i.v./p.o./p.f.t. q. 6 h
Tramadol^112^	263	20	2.6	6.3	Hydrophilic, monitor NRS, CPOT	Urine 30%	50 mg p.o./p.f.t. q. 6 h
Clonidine^113^	230	20	2.9	12	Lipophilic, monitor RASS	Urine 40 %	0.1–0.5 mg p.o./p.f.t. q. 6–12 h
Buproprion^114^	240	84	20	3	Lipophilic	Urine 87 %, feces 10%	100 mg IR p.o./p.f.t. b.i.d.
Trazodone^115^	372	89	0.5	7	Lipophilic, monitor RASS	Urine 70%, feces 21%	12.5–50 mg p.o./p.f.t. q.d. at bedtime
Venlafaxine^116^	277	27	7.5	5	Hydrophilic	Urine 87%,	37.5 mg p.o./p.f.t. q.d.
Fluoxetine^117^	309	95	12	24–96	Lipophilic	Urine	10–20 mg p.o./p.f.t. q.d.
Paroxetine^118^	329	93	8.7	21	Lipophilic	Urine 64%, feces 36%	10–20 mg p.o./p.f.t. q.d.
Sertraline^119^	306	98	20	26	Lipophilic	Urine 40%, feces 40%	25–50 mg p.o./p.f.t. q.d.
Amitriptyline^120^	277	>90	18–22	13–36	Lipophilic	Urine	10–25 mg p.o./p.f.t. q.d.
Nortriptyline^121^	263	86	21	26	Lipophilic	Urine	10–25 mg p.o./p.f.t. q.d.
Quetiapine^122^	384	83	10	6	Lipophilic, monitor RASS	Urine 73 %, feces 20%	Sedation: 25 mg p.o./p.f.t. q.d. – q. 12 h Bipolar: 100–200 mg p.o./p.f.t. q evening
Risperidone^123^	410	90	1–2	20	Lipophilic, monitor RASS	Urine 70%, feces 14%	1–2 mg p.o./p.f.t. q.d.
Aripiprazole^124^	448	>99	4.9	75	Lipophilic, monitor RASS	Feces 55%, Urine 25%	10–15 mg p.o./p.f.t. q.d.
Olanzapine^125^	312	93	14	30	Lipophilic, monitor RASS	Urine 57%, feces 30%	2.5–5 mg p.o./p.f.t. q.d. 2.5–5 mg i.v./i.m. q. 2–4 h p.r.n.

Pharmacokinetic parameters of protein binding, volume of distribution, and half-life summarized in this table were obtained from product insert information, largely derived from healthy adults. CCRT, continuous renal replacement therapy; CPOT, Critical Care Pain Observation Tool; i.m., intramuscularly; IR, immediate release; i.v., intravenously; MW, molecular weight; NRS, numeric rating scale; PK/PD, pharmacokinetic/pharmacodynamic; p.f.t., per feeding tube; p.r.n., as needed; q., every; q.d., every day; RASS, Richmond Agitation−Sedation Scale.

a Suggestion based on opioid-naive patient. Lowest effective dose should be used.

b Feeding tube administration varies by manufacturer. Please check manufacturer information prior to trialing.

c Assuming a 70-kg patient.

d Suggestion for psychotropic medication based on starting doses for intensive care unit sedation purposes only. Higher doses may be indicated based on past medical history, concomitant underlying conditions, or concomitant use of inducer.

The most important factor affecting medication clearance via CRRT circuit is protein binding. Intravascular proteins to which medications may bind include albumin, α-1 acid glycoprotein, and lipoproteins. Albumin binds acidic drugs such as phenytoin, warfarin, digoxin, naproxen, ceftriaxone, lorazepam, and valproic acid. α-1 125Glycoprotein binds basic drugs such as lidocaine, propranolol, and tricyclic antidepressants. Advanced age, sex, chronic critical illness, and malnutrition may predispose patients to chronically low protein concentrations within the body prior to ICU admission.^126^, ^127^, ^128^ During an acute stress, an acute-phase response occurs in the body, resulting in rapid and drastic decreases in albumin and α-1 glycoprotein, with fluctuations in lipoproteins occurring as well.^129^^,^^130^ The fraction of medications that is unbound, also considered the active form of medication, is available for metabolism and elimination and is more likely to be removed by CRRT. Protein-bound medication complexes are likely to accumulate in renal dysfunction and are less likely to be significantly removed via CRRT circuits, given their size.^12^ Examples of highly protein-bound anticonvulsants and psychotropic medications include phenytoin, carbamazepine, and valproic acid. Meanwhile, anticonvulsants and psychotropic medications such as levetiracetam, gabapentin, and phenobarbital are significantly removed via CRRT. Of note, because protein binding fluctuates with critical illness, the proportion of free drug may also fluctuate. Where available, we recommend measuring free drug concentrations for drugs such as valproic acid or phenytoin to mitigate the risk for adverse effects. An SC may be used to evaluate the ratio of medication concentration in the effluent compared to concentrations in the plasma, in efforts to evaluate the extent of drug removal via CRRT.^131^^,^^132^ Of note, in the setting of overdose, protein binding sites may be saturated and the unbound proportion of drug may be significantly higher, allowing for greater clearance via the CRRT circuit.

Another important factor influencing drug clearance via CRRT is the volume of distribution (V_d_).^131^^,^^133^ Although V_d_ is affected by drug charge and lipophilicity, in general, highly protein-bound medications tend to have smaller V_d_, to be distributed in the plasma, to have shorter half-lives, and to undergo greater removal of unbound drug by CRRT.^131^^,^^132^ Conversely, medications with lower protein binding tend to have larger V_d_, are distributed within extravascular tissue compartments, have longer half-lives, and are less readily removed by CRRT.^131^^,^^132^ The V_d_ may be larger at baseline in patients with chronic comorbidities such as chronic heart failure,^134^ chronic liver failure,^135^ or obesity.^136^ However, in the setting of acute critical illness, a high proportion of patients experience increases in V_d_ of 2-fold or greater^137^ due to the presence of endothelial damage, increased capillary permeability, fluid resuscitation in the setting of shock states, mechanical ventilation, extracorporeal circuits (e.g., extracorporeal membrane oxygenation, cardiopulmonary bypass, plasma exchange), or injuries affecting insensible losses (e.g., burn injuries).^138^^,^^139^ Therefore, most medications that have low V_d_ normally may have larger V_d_ in this setting, resulting in altered medication efficacy. Common examples of anticonvulsants, analgesics, and psychotropic medications with large V_d_ include benzodiazepines, barbiturates, propofol, and fentanyl. For these medications, larger loading doses are recommended to ensure decreased time to therapeutic efficacy. Maintenance doses, however, do not tend to require large adjustments for CRRT.

Related to V_d_, drug lipophilicity may also affect drug clearance via CRRT. The higher the lipophilicity of a medication, the more likely the medication will distribute and accumulate in tissues, have central nervous system side effects, and have a prolonged contact-sensitive half-life, ultimately affecting the amount of free drug available within the intravascular space for CRRT removal. Many of the analgesics, anticonvulsants, and psychotropic medications have high lipophilicity, which supports their often-intended efficacy within the central nervous system. Depending on body habitus and fat stores, loading doses are generally recommended to reach therapeutic concentrations more rapidly. In the setting of medication overdose, it is still feasible to remove lipophilic medications by CRRT. For drugs with multicompartment distribution, saturation of extravascular compartments will lead to higher concentrations in the plasma and greater removal of drug by extracorporeal methods. Removal of drug from vascular compartments will lead to re-equilibration between vascular and tissue compartments, slowly clearing drug accumulation from tissue compartments.

Finally, medication removal via CRRT may be affected by the molecular weight (MW) of a medication. Medications with an MW of less than or equal to 2000 Daltons may be readily removed by all modalities of CRRT with the advent of high-flux hemofilters. All analgesics, anticonvulsants, and psychotropic medications are small-molecule drugs that have an MW of less than 500 Daltons, indicating the MW will not hinder removal by CRRT. Medications greater than 15,000 Daltons in weight are considered too large to be removed by CRRT circuits at this time. For medications between 2000 and 15,000 Daltons in weight, the degree of clearance depends on the modality of CRRT used. In the setting of CVVHD, the diffusion rate across the CRRT filter dictates drug clearance, with lower-MW medications diffusing more rapidly, and therefore cleared to a greater extent, than higher-MW medications. In the setting of CVVH, and partly with CVVHDF, convection ensures relatively stable rates of medication removal, irrespective of weight, for MWs between 2000 and 15,000 Daltons. For these modalities, however, ultrafiltration flow rates, use of a pre-filter replacement fluid with CVVH, and the ratio of dialysis inflow to effluent flow rate for CVVHDF may affect drug clearance.

### Practical Considerations to Guide Drug Dosing

There are many practical considerations integral to determining appropriate drug dosing in the setting of CRRT. Prior to adjusting medication dosing for CRRT, 1 of the most important practical considerations is the indication for CRRT. In the setting of AKI, initially drugs should be dosed based on CRRT prescription, the severity of the clinical situation, and a risk-versus-benefit evaluation of drug efficacy versus toxicity. Subsequently, reassessment of dosing is based on estimates of the delivered CRRT clearance and monitoring of treatment goals. In the absence of drug concentrations to guide dosing, monitoring of laboratory test results and clinical status for signs or symptoms of toxicity is recommended especially in situations necessitating aggressive dosing. Drug concentrations from medications administered with similar properties can be used to extrapolate potential clearance of similar drugs administered to the patient.

Although CRRT in the setting of AKI is most common, other indications for CRRT exist, such as volume overload, acidosis, electrolyte disturbances, or to remove toxins. In these circumstances, the degree of organ dysfunction present prior to initiation of CRRT influences the degree of drug clearance via renal and nonrenal routes. Unfortunately, appropriate dosing of medications in these circumstances is highly uncertain, given the presence of even fewer data to guide treatment and the high variability in patient PK/PD characteristics. However, aggressive dosing strategies would be warranted under these circumstances, to ensure appropriate therapeutic efficacy. Generally, medications should be dosed at least as aggressively as they would be dosed immediately preceding initiation of CRRT. Further dose increases, however, may be warranted, depending on the CRRT prescription, expected medication clearance by CRRT, and risk-versus-benefit evaluation of drug efficacy versus toxicity. Strategies that we will discuss next, such as use of loading doses, therapeutic drug monitoring, clinically significant pharmacodynamic parameters, target-site penetration, and context-sensitive half-life, should all be considered in efforts to ensure that underdosing of medications does not occur.

For highly lipophilic medications, ensuring appropriate loading dose administration is imperative in order to evaluate therapeutic efficacy in a timely manner. This property pertains to most analgesic, anticonvulsant, and psychotropic medications. In the setting of kidney dysfunction, most clinicians hesitate to administer larger loading doses for fear of overdosing; however, use of loading doses is crucial, given prolonged dosing intervals and longer time to achieve steady-state therapeutic efficacy. In the setting of a continuous infusion of analgesic or sedative medication, liberal use of loading doses and intermittent bolus doses can not only achieve therapeutic concentrations, but can potentially mitigate risk of overaccumulation associated with aggressive up-titration and dose stacking of continuous infusions.

Monitoring concentrations may be helpful, if available, to support therapeutic efficacy and to mitigate the risk of medication toxicities. Only a small fraction of medications may have drug concentrations available for clinicians within a reasonable and meaningful timeframe to adjust therapy. However, given the fluctuations in PK/PD parameters throughout the course of critical illness, greater use of therapeutic drug monitoring, when available, is recommended to evaluate medication efficacy in critically ill patients. This can be especially valuable when evaluating the safety and efficacy of medications with narrow therapeutic indices. However, as previously mentioned, drug concentrations of medications with broad therapeutic indices may still be helpful to identify underdosing and to inform clearance estimation of other medications of interest with similar PK/PD properties that do not have therapeutic drug monitoring. Table 3 summarizes common medications with available therapeutic drug monitoring that can be used in practice. Given that many of the analgesics, anticonvulsants, and psychotropic medications have multicompartment kinetics, drug concentrations may be helpful post−loading dose to characterize changes in Vd, before steady state to characterize early estimates of clearance, and most importantly, after steady state receptor activity is achieved, to monitor efficacy, accumulation, and toxicity.

**Table 3:** Common medications with available therapeutic drug monitoring

Psychotropic medications	Antibiotics	Immunosuppressants	Other
Carbamazepine	Vancomycin	Cyclosporine	Digoxin
Phenobarbital	Gentamicin	Everolimus	Theophylline
Phenytoin	Amikacin	Tacrolimus	Voriconazole
Valproic Acid	Tobramycin	Sirolimus	Posaconazole

Many medications have PD therapeutic efficacy parameters that may support or guide dose adjustments. For example, in the setting of analgesics, the numeric rating scale (NRS) in patients able to self-report their pain, or the Critical Care Pain Observation Tool (CPOT) in patients unable to self-report pain, may be used to evaluate the presence of pain in critical illness and may support appropriate medication dose adjustments.^140^^,^^141^ For example, the Richmond Agitation−Sedation Scale (RASS) may be used to evaluate depth of sedation and to support sedative medication adjustments, and electroencephalography may be used for antiepileptic medications to evaluate therapeutic efficacy.^142^, ^143^, ^144^ These classes of medications include high-risk drugs with narrow therapeutic indices. Oversedation has been associated with adverse effects including acute delirium, longer duration of mechanical ventilation, longer ICU and hospital length of stay, posttraumatic stress disorder, chronic depression, higher healthcare costs, and higher mortality in critically ill patients.^19^, ^20^, ^21^, ^22^, ^23^, ^24^, ^25^, ^26^, ^27^, ^28^ Meanwhile, underdosing of analgesics and psychotropic medications has been associated with poor patient outcomes, including but not limited to cardiac instability, respiratory compromise, delirium, and medication withdrawal.^18^^,^^145^, ^146^, ^147^, ^148^ In general, target site penetration of analgesic, anticonvulsant, or psychotropic medications may also be altered in the setting of critical illness, and therefore therapeutic efficacy via any PD strategies should be used to support therapy adjustments as needed. Receptor binding affinity may influence time to therapeutic effect as well as rate of clearance via CRRT circuit; therefore, biological half-life (separate from kinetic half-life) should be considered. Finally, for all medications with a large V_d_ and high lipophilicity, the context-sensitive half-life should be kept in mind. The decision regarding which half-life to monitor should be based on drug properties, with an emphasis on biologic and context-sensitive half-life estimation in these particular drug classes whenever possible, given that the therapeutic efficacy and duration of effects of medications will be highly dependent on cumulative drug exposure. Although these half-lives cannot be easily calculated with a standard equation, primary literature and package inserts may be used for general guidance on estimate half-life and duration of effects. Closer monitoring of these medications is important.

### Gaps in Current Research

Unfortunately, there continue to be major gaps in our current knowledge of analgesic, anticonvulsant, and psychotropic medication clearance in critically ill patients on CRRT. Table 4 summarizes current drug adjustment recommendations in CRRT for the most common psychotropic and anticonvulsant mediations. Some of the greatest challenges to dosing adjustment recommendations include lack of data, large variability in design, variability in reported pharmacokinetic parameters, lack of availability of sieving coefficients, limited patient populations, and limited clinical circumstances reported in the published literature to date. Under those circumstances, caution should be exercised when applying existing data to other populations.

**Table 4:** Drug adjustment recommendations in continuous renal replacement therapy

Medication	CRRT mode		
	CVVH	CVVHD	CVVHDF
**Analgesics**			
Oxycodone^70^	No data on CRRT; 33−50% of usual initial dosing have been recommended in renal impairment		
Hydromorphone^71^	No data on CRRT; 25−50% of usual initial dose + extend the dosing interval by 25−50% (expert opinion)		
Morphine^72^^,^^73^	Other opioids are preferred (eg, hydromorphone, fentanyl) due to more reliable pharmacokinetic profiles (expert opinion). Morphine is not significantly removed by CVVH or CVVHD,^73^ and the extent of M3G and M6G removal is unknown		
Fentanyl^74^	No data on CRRT; Use lowest effective dose with gradual titration and close monitoring of response and adverse effects (expert opinion). Dose for GFR 10−50 (expert opinion)		
Methadone^75^	No data on CRRT; Dose for GFR 10−50 (expert opinion)		
**Anticonvulsants**			
Valproic acid^76^, ^77^, ^78^	Removed by CVVH^36^, ^77^	No data	Removed by CVVHDF^78^
Carbamazepine^79^, ^80^, ^81^	No data	Removed by CVVHD^80^^a^	Removed by CVVHD^81^
Levetiracetam^82^, ^83^, ^84^, ^85^	Cleared by CVVH^83^^,^^85^	No data	Removed by CVVHDF^83^^,^^84^
Phenytoin^86^^,^^87^	Approximately 30% cleared by CVVH^87^	No data	No data
Lacosamide^88^, ^89^, ^90^	Significant removal by CVVH^89^^,^^90^	No data	No data
Topiramate^91^^,^^92^	No data	No data	Removed by CVVHDF^92^
Lorazepam^93^^,^^94^	Not efficiently removed by CVVH^94^	No data	No data
Midazolam^94^^,^^95^	Unconjugated 1-hydroxymidazolam not effectively removed; 1-hydroxymidazolamglucuronide effectively removed; SC = 0.45^94^	No data	No data
Clonazepam^96^	No data on CRRT		
Diazepam^97^	No data on CRRT		
Pentobarbital^98^^,^^99^	Removed by CVVH based on case report where less was cleared during CVVH interruption^99^	No data	No data
Phenobarbital^100^, ^101^, ^102^, ^103^	No data	Removed by CVVHD^102^^,^^103^	Removed by CVVHDF^101^
Gabapentin^104^^,^^105^	No data	Cleared by CVVHD^105^	No data
Pregabalin^106^	No data on CRRT		
**Additional Sedatives and Psychotropic Medications**			
Propofol^107^	No data on CRRT; dose for GFR 10−50 mL/min/1.73m^2^(expert opinion)		
Dexmedetomidine^108^	No data on CRRT		
Ketamine^109^	No data on CRRT; dose for GFR 10−50 mL/min/1.73m^2^(expert opinion)		
Acetaminophen^110^^,^^111^	Removed by CVVH^111^	Removed by CVVHD^111^	No data
Tramadol^112^	No data on CRRT		
Clonidine^113^	No data on CRRT; dose for GFR 10−50 mL/min/1.73m^2^(expert opinion)		
Buproprion^114^	No data on CRRT		
Trazodone^115^	No data on CRRT		
Venlafaxine^116^	No data on CRRT; dose for GFR 10−50 mL/min/1.73m^2^(expert opinion)		
Fluoxetine^117^	No data on CRRT; dose for GFR 10−50 mL/min/1.73m^2^(expert opinion)		
Paroxetine^118^	No data on CRRT; dose for GFR 10−50 mL/min/1.73m^2^(expert opinion)		
Sertraline^119^	No data on CRRT		
Amitriptyline^120^	No data on CRRT		
Nortriptyline^121^	No data on CRRT		
Quetiapine^122^	No data on CRRT		
Risperidone^123^	No data on CRRT		
Aripiprazole^124^	No data on CRRT		
Olanzapine^125^	No data on CRRT		

CRRT, continuous renal replacement therapy; CVVH, continuous venovenous hemofiltration; CVVHD, continuous venovenous hemodialysis; CVVHDF, continuous venovenous hemodiafiltration; GFR, glomerular filtration rate.

a Drug removal is shown in the setting of overdose. The proportion of free drug available for removal in this setting of saturated receptors and all proteins bound may differ from that removed with more conservative drug exposure during critical illness.

As advancements are made in CRRT circuits, extrapolation from published data on drug clearance in outdated CRRT modalities or techniques may lead to mis-dosing of medications. Furthermore, alterations in prescription need to be taken into account, and are not being studied in a systematic fashion at this time. Future studies evaluating differences in medication clearance depending on effluent flow rate will be beneficial to more accurately guide dosing in CRRT. As contemporary advancements in CRRT technology occur, drug dosing information needs to be re-evaluated to ensure that clinical dosing guidance is still accurate.

At this time, there are certainly underrepresented drug classes in the literature evaluating the impact of CRRT on drug clearance. A greater number of studies have been published on antibiotic drug clearance, with limited data on antiepileptic drugs or other classes of medications. A great proportion of medications used in the critical care setting may remain influenced by CRRT removal, but have yet to be evaluated for clearance. Use of the concepts discussed in this review may support decisions regarding drug dosing in the interim. However, future studies of different classes of medications are much needed, to support clinicians.

Finally, an important limitation to the current literature is the paucity of generalizable data. Current data, even in critically ill patients, may not always be accurately applied to all critically ill patients, because of fluctuations in host pharmacokinetics in certain states of critical illness. For example, data on drug clearance in the setting of drug overdose, when protein-binding sites are saturated and the unbound proportion of drugs may be higher, may overestimate CRRT clearance in patients with critical illness or lower total circulating drug concentrations. Lower proportions of unbound drug may result in less clearance of administered drug. Therefore, application of drug dose adjustments made based on CRRT clearance from overdose reports has the potential to result in underdosing other populations of critically ill patients when greater proportions of drug−protein complexes exist. Similarly, medications that are highly lipophilic may be removed by CRRT circuit to a different extent in patients who are morbidly obese versus cachectic. Dosing adjustments suggested from smaller studies of patients on CRRT may not be generalizable to patients with different host or pharmacokinetic characteristics.

## Conclusion

This article summarizes the most pertinent patient-related, medication-related, and CRRT prescription characteristics to consider in efforts to support drug dosing in CRRT. We also summarize the current studies published supporting CRRT dosing of analgesics, anticonvulsants and psychotropic medications, along with gaps in the current literature. Future studies are needed to support more accurate dosing of anticonvulsants, analgesics, and psychotropic medications in a broader range of clinical circumstances and with contemporary CRRT technologies.

## Disclosure

Both authors declared no competing interests.
